# Supplementary material for: Content-rich biological network constructed by mining PubMed abstracts
Source: BMC Bioinformatics. 2004 Oct 8;5:147. doi: 10.1186/1471-2105-5-147 (PMC528731; doi:10.1186/1471-2105-5-147)
Supplement: Additional File 2 — The original results of the above study (non-essential files are deleted to keep the file size under the limit set by BMC bioinformatics). [file 1471-2105-5-147-S2.bz2 › chilibotAdditionalFile2/dip20/362ID8558064E5750/html/IL8_FY.html]

 


 **IL8** and **FY** 
  
Found 25 abstracts in PubMed, retrieved 20.  
 

 What does Google say? 
 PDF only 
| .edu only 

---

**Interactive relationship** (e.g. stimulation, inhibition, etc)

**Neutral relationship**- IL 8  [ **IL8** ]  single chain homodimers and heterodimers interactions with chemokine receptors CXCR1, CXCR2, and DARC  [ **FY** ] .  Ref: 9070443 Protein Sci, 1997
- Crosslinked single chain dimers bind to both IL 8  [ **IL8** ]  neutrophil receptors CXCR1 and CXCR2 as well as to DARC  [ **FY** ] , as does a double disulfide linked dimer with no peptide linker.  Ref: 9070443 Protein Sci, 1997
- The monomeric variants of IL 8  [ **IL8** ]  bind the erythrocyte chemokine receptor DARC  [ **FY** ] , as well as the neutrophil IL 8  [ **IL8** ]  receptors CXCR1 and CXCR2 with affinities similar to that of wild type IL 8  [ **IL8** ] .  Ref: 9070442 Protein Sci, 1997
- Results showed that supernatants from DARC  [ **FY** ]  ve concentrates contained higher levels of IL 8  [ **IL8** ]  up to days 14 15 of storage compared with DARC  [ **FY** ]  ve RCCs.  Ref: 10925048 Transfus Sci, 2000
- Augmented IL 8  [ **IL8** ]  would directly and or indirectly promote angiogenesis by binding to DARC  [ **FY** ]  and by inducing leukocyte infiltration and activation by binding to CXCR1 and CXCR2.  Ref: 9334359 J Exp Med, 1997
- The red blood cell Duffy antigen receptor for chemokines  [ **FY** ]  serves as a sink for the clearance of chemokines such as interleukin 8  [ **IL8** ]  IL 8  [ **IL8** ]  from the circulation.  Ref: 10964545 Clin Immunol, 2000
- Plasmodium vivax and the related monkey malaria, P. knowlesi, require interaction with the Duffy blood group  [ **FY** ]  antigen, a receptor for a family of chemokines that includes interleukin 8  [ **IL8** ] , to invade human erythrocytes.  Ref: 8046329 J Exp Med, 1994
- Biopanning of phage peptide libraries with an anti Fy6 monoclonal antibody led to the identification of the motif Phe22 Glu23, the mutation of which altered the binding of both anti Fy6 and chemokines interleukin 8  [ **IL8** ] , MGSA, RANTES regulated on activation normal T cell expressed to DARC  [ **FY** ]  transfectants.  Ref: 9195930 J Biol Chem, 1997
- DARC  [ **FY** ]  belongs to a family of heptahelical chemokine receptors that includes specific IL 8RA and shared IL 8RB IL 8  [ **IL8** ]  receptors.  Ref: 7592830 J Biol Chem, 1995
- The Duffy Antigen Receptor for Chemokines  [ **FY** ]  DARC  [ **FY** ]  belongs to a family of erythrocyte chemokine receptors that bind C X C and C C chemokines such as interleukin 8  [ **IL8** ]  IL 8  [ **IL8** ] , monocyte chemoattractant protein 1 MCP 1 and regulated on activation, normal T cell expressed and secreted RANTES, but not macrophage inflammatory protein 1 alpha MIP 1 alpha or MIP 1 beta.  Ref: 9921412 Biomed Pharmacother, 1998
- Scatchard analysis demonstrated that a chimeric DARC  [ **FY** ]  receptor, composed of the E1 domain of DARC  [ **FY** ]  and the predicted hydrophobic helices and loops of interleukin 8RB IL 8RB, bound IL 8  [ **IL8** ] , and MGSA with KD values almost identical to the wild type receptors and bound a repertoire of C X C and C C chemokines characteristic of DARC  [ **FY** ] .  Ref: 8558064 J Leukoc Biol, 1996
- Binding of IL 8  [ **IL8** ]  to both rheumatoid and non rheumatoid synovia was significantly reduced in the presence of anti DARC  [ **FY** ]  Fy3 and Fy6 monoclonal antibodies.  Ref: 12081195 J Pathol, 2002

**Non-interactive relationship** (e.g. studied together, co-existance, homology, etc.)

- The involvement of DARC  [ **FY** ]  in chemokine binding was studied by incubating sections with blocking antibodies to DARC  [ **FY** ]  Fy3 and 6, to find out if these reduced 125I IL 8  [ **IL8** ]  binding.  Ref: 12081195 J Pathol, 2002
- Although previous studies suggested that interleukin 8  [ **IL8** ]  bound either directly to or close to the Fy6 epitope, the ability of  **Fy** , 6 rhesus monkey cells to adsorb interleukin 8  [ **IL8** ]  suggests that the interleukin 8  [ **IL8** ]  receptor is distinct from Fy6, Fy1, and Fy2.  Ref: 8693506 Transfusion, 1996
- Human  **Fy** , 6 red cells bound minimal interleukin 8  [ **IL8** ] .  Ref: 8693506 Transfusion, 1996
- The new variant has been characterized by its binding affinity to IL 8  [ **IL8** ]  receptors A and B and the erythrocyte receptor DARC  [ **FY** ] .  Ref: 9141135 Proteins, 1997
- Rhesus monkey cells lacking Fy1, Fy2, and Fy6 were able to adsorb interleukin 8  [ **IL8** ]  in a manner similar to  **Fy** , 6 or  **Fy** , 6 primate cells.  Ref: 8693506 Transfusion, 1996
- We also determined serum tumor necrosis factor alpha TNF alpha, the phenotypes of the IL 8  [ **IL8** ]  binding Duffy blood group  [ **FY** ]  antigen receptor and the von Willebrand factor vWF plasma levels.  Ref: 12516654 Am J Reprod Immunol, 2002
- **Fy** , 6 or  **Fy** , 6 owl monkey, squirrel monkey, human red cells adsorbed interleukin 8  [ **IL8** ]  over a wide range of concentrations 125 1000 pg mL.  Ref: 8693506 Transfusion, 1996
- Alanine scanning mutagenesis of the charged amino acids of melanoma growth stimulating activity MGSA was used to identify specific residues that are involved in binding to the human erythrocyte Duffy antigen chemokine receptor  [ **FY** ]  DARC  [ **FY** ]  and to the type B interleukin 8  [ **IL8** ]  receptor IL 8RB on neutrophils.  Ref: 7744785 J Biol Chem, 1995
- The lack of the chemokine binding was not likely to be due to the lack of glycosylation of the  **Fy**  GPA, since IL 8  [ **IL8** ]  was effectively bound to de N glycosylated erythrocytes.  Ref: 10873668 Biochem Biophys Res Commun, 2000
- mRNA for IL 8  [ **IL8** ]  binding chemokine receptors CXCR1, CXCR2, and the Duffy antigen receptor for chemokines  [ **FY** ]  DARC  [ **FY** ]  were found in all astrocytoma grades by reverse transcription PCR analysis.  Ref: 9334359 J Exp Med, 1997
- We have shown previously that chemokine receptors including the interleukin 8  [ **IL8** ]  receptor B and the Duffy blood group  [ **FY** ]  antigen are expressed on subsets of neurons in various regions of the adult nervous system.  Ref: 9024623 Curr Biol, 1997
- The serum levels of IL 8  [ **IL8** ]  and monocyte chemotactic peptide MCP 1, two DARC  [ **FY** ]  binding chemokines, were investigated and related to this polymorphism.  Ref: 12739041 Rheumatol Int, 2003
